# Concomitant prednisone may alleviate methotrexate side-effects in rheumatoid arthritis patients

**DOI:** 10.1186/s41927-023-00331-0

**Published:** 2023-05-17

**Authors:** Matthijs S. van der Leeuw, Janneke Tekstra, Jacob M. van Laar, Paco M. J. Welsing

**Affiliations:** Department of Rheumatology and Clinical Immunology, University Medical Center Utrecht, Heidelberglaan 100, Utrecht, 3584CX The Netherlands

**Keywords:** Rheumatoid arthritis, Methotrexate, Prednisone, Glucocorticoids, Drug safety

## Abstract

**Objectives:**

To evaluate whether addition of low-moderate dose prednisone to methotrexate (MTX) treatment can alleviate common MTX side-effects in rheumatoid arthritis (RA) patients.

**Methods:**

We performed a post-hoc analysis of the CAMERA-II trial which randomized (1:1) 236 early DMARD and prednisone naive RA patients to treatment with MTX + prednisone 10 mg daily, or MTX monotherapy during two years. MTX dose was increased using a treat-to-target approach. We used Generalized Estimating Equations to model the occurrence of common MTX side-effects and of any adverse event over time, controlling for disease activity and MTX dose over time and other possible predictors of adverse events. To assess whether a possible effect was prednisone-specific, we performed the same analysis in the U-ACT-EARLY trial, in which the addition of tocilizumab (TCZ) to MTX was compared to MTX monotherapy in a comparable setting.

**Results:**

MTX side-effects were reported at 5.9% of visits in the prednisone-MTX group, compared to 11.2% in the MTX monotherapy group. After controlling for MTX dose and disease activity over time, treatment duration, age, sex, and baseline transaminase levels, addition of prednisone significantly decreased the occurrence of MTX side-effects (OR: 0.54, CI: 0.38–0.77, *p* = 0.001). Specifically, the occurrence of nausea (OR 0.46, CI: 0.26–0.83,  *p* = 0.009)) and elevated ALT/AST (OR 0.29, CI: 0.17–0.49, *p*  < 0.001) was decreased. There was a trend towards fewer overall adverse events in the prednisone-MTX arm (OR: 0.89, CI: 0.72–1.11, *p* = 0.30). No difference in MTX side-effects was found between TCZ-MTX and MTX monotherapy in U-ACT-EARLY (OR 1.05, CI: 0.61–1.80, *p*  = 0.87).

**Conclusion:**

Addition of 10 mg prednisone daily to MTX treatment in RA patients may ameliorate MTX side-effects, specifically nausea and elevated ALT/AST.

**Supplementary Information:**

The online version contains supplementary material available at 10.1186/s41927-023-00331-0.

## Keypoints

Concurrent use of 10 mg prednisone may ameliorate MTX side-effects, specifically nausea and elevated ALT/AST.

**Supplementary Information:**

The online version contains supplementary material available at 10.1186/s41927-023-00331-0.

## Introduction

After a diagnosis of rheumatoid arthritis (RA) has been made, treatment with methotrexate (MTX) should be initiated as soon as possible, increasing the dose up to 25-30 mg/week until the disease is in remission. When remission is not reached or lost later on, an additional Disease Modifying Anti Rheumatic Drug (DMARD) is usually added to the MTX treatment. MTX thus serves as an anchor drug in the treatment of RA [1]. However, MTX toxicity is common and can limit the maximum usable dose or lead to discontinuation. The most frequently reported side-effects are nausea, vomiting, abdominal pain, elevated liver enzymes, loss of appetite and ulcers of the digestive tract [2]. Less common but severe side-effects include pancytopenia, pneumonitis and decreased renal function. Overall, side-effects were found to lead to complete discontinuation in 10–37% of cases within 12.7 years of treatment, thereby hampering effective treatment of RA [3].

Many RA patients also receive concomitant treatment with prednisone since this significantly and quickly reduces disease activity, and can also reduce radiographic progression of joint damage, as has been shown in several double blinded randomized trials [4]. In one of these trials (CAMERA-II) prednisone (10 mg daily) in addition to MTX was compared to MTX monotherapy for a 2-year period. Besides significantly reduced disease activity and radiographic progression in the prednisone arm after 2 years, data from this trial also seemed to suggest that concurrent use of prednisone might alleviate some of the common side-effects of MTX compared to MTX monotherapy [5]. If this is true, then concomitant use of low-moderate dose prednisone might improve MTX retention rates and increase the maximum tolerable dose, next to being an effective combination treatment strategy. However, due to the dynamic dosing of MTX and the effectiveness of additional prednisone, MTX dose and disease activity over time differed between treatment arms, which may influence the occurrence/experience of side-effects. To investigate this this possible effect in more detail, we performed a post-hoc analysis on data from this randomized trial, controlling for these confounding factors over time.

## Methods

### Patients and intervention

We used data from the CAMERA-II trial, in which 236 early DMARD and prednisone naive RA patients were randomized 1:1 to treatment with MTX + prednisone 10 mg daily, or MTX + placebo during two years [5]. Initial MTX dose was 10 mg weekly in combination with folic acid 0.5 mg daily (except on the day of MTX intake). At monthly visits, treatment was adjusted according to the treat-to-target principle, increasing MTX dose by 5 mg, until either the treatment target or the maximum (30 mg, or maximum tolerable) dose was reached. If remission was still not reached, MTX was administered subcutaneously, and as a next step adalimumab was added (with MTX and prednisone/placebo being continued unchanged). Treatment with prednisone or placebo was double blind, and the dose was not tapered during the follow up period. In total 4,608 visits (in 236 patients) at which information was available on MTX dose and adverse events (AE), were used for analysis in the current post-hoc analysis.

### Outcomes

We assessed all MTX side-effects with a reported incidence > 10% according to the Netherlands Pharmacovigilance Centre (Lareb) [2] which consisted of oral ulcers, dyspepsia, abdominal pain, loss of appetite, nausea, vomiting and elevation of Alanine Transaminase (ALT) and Aspartate Transaminase (AST). The primary endpoint for the analysis was a composite endpoint defined as the occurrence of any of these MTX side-effects during the preceding period at each visit. As such the analysis accounted for both the occurrence and the duration of side-effects because they were reported multiple times by a patient if they were persistent. Secondary endpoints were the occurrence of most common individual MTX side-effects: nausea, and elevated AST or ALT defined as higher than two times the upper limit of normal. To assess possible AEs related to prednisone we also analysed the occurrence of any AE (irrespective of its relation to MTX) and any AE excluding MTX side-effects.

### Analysis

To evaluate these endpoints, we used logistic regression by Generalized Estimating Equations with an unstructured correlation structure accounting for the repeated observations within patients. In this model we corrected for time, MTX dose used during the preceding period and Disease Activity Score 28 based on erythrocyte sedimentation rate (DAS28) at a visit over time. We also explored the effect of age, sex, presence of rheumatoid factor, use of adalimumab, baseline ALT/AST, number of clinic visits and a non-linear (quadratic) effect for time (since it was deemed likely that the effect of time was non-linear, e.g. due to habituation). To arrive at a final model, we used a stepwise model building strategy, starting with a full model and stepwise excluding covariates that did (1) not improve the model fit (reduction in corrected Quasi-likelihood under Independence Model Criterion (QICC) of 3 or less), (2) were not significantly associated with the occurrence of MTX side-effects (*p* < 0.05), and (3) did not alter the Odds Ratio (OR) for the effect of prednisone by more than 5%. DAS28 and MTX dose over time were retained in the model irrespectively, because of their clinical relevance. Finally, in case a statistically significant effect of prednisone was found, we also explored modification of this effect by other covariates that were predictive of MTX side-effects, using an interaction term. In case the p-value for an interaction term was < 0.1, effect modification was deemed possible and a stratified analysis for this modifier was performed. Relative Risks (RR) were calculated for side-effects that occurred at > 10% of visits.

To evaluate whether any effect on the occurrence of MTX side-effects can be attributed to prednisone, and is not a general effect of concomitant immunosuppressive therapy, we performed the same analysis in data from the U-ACT-EARLY trial [6]. This was a double blind trial in which DMARD naive RA patients from a similar population (i.e. the same country and region) were randomized to either treatment with MTX with additional tocilizumab (8 mg/kg every 4 weeks; N = 106), MTX + placebo (N = 104) or TCZ + placebo. The latter arm was not evaluated in the current study. MTX step-up treatment as well as the treatment target was comparable to CAMERA-II. Hydroxychloroquine was added to treatment if the target was not reached with the maximum dose of MTX. Only visits until 6 months follow-up (N = 1,227) were used, since from this point tocilizumab (TCZ) could be added to the MTX monotherapy group in case remission was not reached (and the treatment strategies were thus equal between arms).

All analyses were performed using SPSS software version 26.0.0.1.

## Results

Table 1 shows the general patient characteristics, medical history and occurrence of side-effects of the CAMERA-II and U-ACT-EARLY trials. Mean DAS28 scores over time were lower in the arms where prednisone or TCZ was added to MTX (2.4 and 2.0 respectively) compared to the corresponding MTX monotherapy arms (3.0 and 3.7 respectively). Mean weekly MTX dose over time was also lower (16.0 and 11.8 mg versus 20.5 and 19.9 mg) in the combination therapy arms.

**Table 1:** General patient characteristics and occurrence of side-effects

	CAMERA-II		U-ACT-EARLY (first 6 months)	
	MTX + prednisone (N = 117; 2187 visits)	MTX Mono (N = 119; 2421 visits)	MTX + TCZ (N = 106; 622 visits)	MTX Mono (N = 108; 605 visits)
Female sex	70 (60%)	72 (61%)	65 (61%)	69 (64%)
RF positive	64 (55%)	73 (61%)	75 (71%)	86 (80%)
Mean age in years (SD)	54 (14)	53 (13)	53 (12)	53 (13)
Mean DAS28^a^ during follow-up (SD)	2.4 (1.3)	3.0 (1.5)	2.0 (1.2)	3.7 (1.4)
Mean MTX^b^ dose in mg during follow-up (SD)	16.0 (8.2)	20.5 (8.0)	11.8 (8.0)	19.9 (7.9)
Adalimumab added	16 (14%)	42 (35%)	–	–
HCQ^c^ added	–	–	23 (21%)	45 (42%)
Discontinued study due to adverse events	16 (14%)	20 (17%)	3 (3%)	1 (1%)
*Medical history at baseline*				
Cardiovascular disease	6 (5%)	6 (5%)	10 (9%)	11 (10%)
Diabetes	2 (2%)	5 (4%)	6 (6%)	3 (3%)
Chronic pulmonary disease	6 (5%)	5 (4%)	8 (8%)	2 (2%)
Malignancy^d^	1 (1%	1 (1%)	4 (4%)	0 (0%)
Chronic gastro-intestinal disease	1 (1%)	1 (1%)	0 (0%)	1 (1%)
*Side-effects: Number of visits (%) (number of patients)*				
Nausea	50 (2.3%) (22)	152 (6.3%) (43)	16 (2.6%) (15)	26 (4.2%) (25)
Vomiting	7 (0.3%) (2)	8 (0.3%) (6)	3 (0.5%) (3)	6 (1.0%) (6)
Abdominal Pain	13 (0.6%) (12)	17 (0.7%) (12)	3 (0.5%) (3)	11 (1.8%) (11)
GI Ulcer^e^	15 (0.7%) (9)	35 (1.4%) (18)	9 (1.4%) (9)	3 (0.5%) (3)
Loss of appetite	1 (0.0%) (1)	4 (0.2%) (3)	0 (0.0%) (0)	3 (0.5%) (3)
Elevated ALT/AST^f^	48 (2.2%) (20)	84 (3.5%) (34)	16 (2.6%) (16)	15 (2.4%) (14)
Any MTX^b^ side-effect	128 (5.9%) (51)	272 (11.2%) (73)	42 (6.8%) (37)	54 (8.7%) (44)
Any adverse event	483 (21.1%) (96)	647 (26.7%) (104)	241 (38.7%) (97)	219 (35.2%) (93)

^a^Disease Activity Score assessing 28 joints

^b^Methotrexate

^c^Hydroxychloroquine

^d^Inactive malignancy, active malignancy was an exclusion criterion

^e^Gastro-intestinal ulcer

^f^Alanine transaminase/aspartate transaminase

In the CAMERA-II trial, AEs were reported at 21.1% of visits in the prednisone-MTX arm, versus 26.7% in the MTX monotherapy arm. MTX related side-effects were reported at 5.9% of visits in the prednisone-MTX arm compared to 11.2% in the MTX monotherapy arm. Most of this difference could be ascribed to a reduction in the occurrence of nausea (2.3% vs 6.3%) and elevation of ALT/AST (2.2% vs 3.5%). In the U-ACT-EARLY data, MTX side-effects occurred at 7.6% of visits in the TCZ-MTX arm, and at 10.6% in the MTX monotherapy arm.

The final model regarding the effect of the addition of prednisone to MTX on the occurrence of MTX side-effects is displayed in Table 2. The addition of prednisone to MTX statistically significantly decreased the likelihood of any MTX side-effect with an OR of 0.54 (CI: 0.38–0.77, RR: 0.57, CI: 0.41–0.79, *p* = 0.001), independently of the MTX dose and disease activity. Male sex and higher age were found to statistically significantly decrease the likelihood of MTX side-effects. A higher MTX dose (as expected) and higher DAS28 slightly increased the risk for MTX side-effects. Regarding effect modification, male sex was found to decrease the protective effect of prednisone against MTX toxicity (*p*-value of interaction 0.065) and therefore stratified results for the final model are also shown in Table 2.

**Table 2:** Final model for the occurrence of MTX side-effects

A. Overall model			
	OR	95% CI	*P*-value
**Use of prednisone**	**0.54**	**0.38–0.77**	**0.001**
Male sex	0.53	0.37–0.77	0.001
Age (years)	0.98	0.97–0.99	0.001
MTX^a^ dose (mg)	1.03	1.01–1.04	0.001
DAS28^b^	1.05	0.96–1.14	0.307
AST^c^ at baseline (U/L)	1.02	1.00–1.04	0.082
ALT^d^ at baseline (U/L)	1.01	1.00–1.02	0.069
Time (months)	0.93	0.89–0.98	0.004
Time^2^ (months)	1.00	1.00–1.00	0.010
Intercept	0.28	0.13–0.62	0.002

B. Model stratified by sex						
	Males			Females		
	OR	95% CI	*P*-value	OR	95% CI	*P*-value
**Use of prednisone**	**0.84**	**0.46–1.55**	**0.586**	**0.42**	**0.28–0.63**	** < 0.001**
Age (years)	0.97	0.95–0.99	0.007	0.98	0.97–1.00	0.009
MTX^a^ dose (mg)	1.05	1.02–1.07	< 0.001	1.02	1.00–1.03	0.066
DAS28^b^	0.97	0.83–1.13	0.711	1.12	1.02–1.24	0.023
AST^c^ at baseline (U/L)	1.00	0.97–1.04	0.906	1.02	1.00–1.05	0.055
ALT^d^ at baseline (U/L)	1.02	1.01–1.04	0.011	1.00	0.99–1.01	0.567
Time (months)	0.93	0.88–0.98	0.013	0.95	0.90–1.01	0.097
Time^2^ (months)	1.00	1.00–1.00	0.026	1.00	1.00–1.00	0.131
Intercept	0.19	0.04–0.88	0.033	0.25	0.09–0.69	0.007

Bold signify the primary outcome of the analysis

^a^Methotrexate

^b^Disease Activity Score assessing 28 joints

^c^Aspartate transaminase d. Alanine transaminase

Both nausea (adjusted OR 0.46, CI: 0.26–0.83, *p* = 0.009) and elevation of AST/ALT (adjusted OR 0.29, CI: 0.17–0.49, *p* < 0.001)) were significantly reduced in the prednisone-MTX arm. Results again suggested that the protective effect was modified by gender for nausea (*p* = 0.039) but not for elevated AST/ALT (*p* = 0.75) (Additional file 1: Table S1). Regarding the occurrence of any AE, there was a trend towards fewer AEs in the prednisone-MTX arm with an OR of 0.89, but this was not statistically significant (CI: 0.72–1.11, RR: 0.92, CI: 0.78–1.08, *p* = 0.30), with no clear modifying effect of gender (*p* = 0.17). AEs other than MTX side-effects were not more common in the prednisone group (OR: 1.05, CI: 0.83–1.34, RR: 1.04, CI: 0.86–1.25), see Additional file 1: Table S2. Possible prednisone related AEs, such as infections, hypertension and diabetes were not more common in the prednisone-MTX arm (see Additional file 1: Table S3 for an overview of AEs per arm). Cushinoid habitus was not reported by anyone, but the mean weight gain was slightly higher in the prednisone-MTX arm group (2.9 kg) than the MTX monotherapy arm (1.3 kg, *p* = 0.03). No protective effect for MTX toxicity was found for the addition of tocilizumab to MTX (OR 1.05, CI: 0.61–1.80, RR: 1.02, CI: 0.79–1.23, *p* = 0.87), indicating the protective effect to be (at least partly) prednisone specific (Additional file 1: Table S4).

## Discussion

This post-hoc analysis suggests that concomitant use of low-moderate dose prednisone may alleviate MTX related side-effects in RA patients, thereby possibly increasing the maximum tolerable dose and effectiveness of MTX treatment. In fact, the mean MTX dose used was higher in the prednisone-MTX group than in the TCZ-MTX group of the trial analysed as reference, although the same maximum dose was specified for the T2T dose steps. This may underscore the better tolerability of MTX when combined with prednisone. No difference in MTX dose used was found between the MTX monotherapy groups of the trials. The alleviating effect of prednisone could not be explained by confounding factors such as MTX dose or disease activity.

The current analysis did not correct for use of NSAIDs (since this data was not systematically collected), which might have differed between arms due to a difference in disease activity. This could have mainly influenced the rates of nausea, and less probably elevation of ALT/AST since this is a less common side-effect of NSAIDs (< 0.1%) than the rates found in this study [7]. Importantly, there was a trend of fewer overall AEs in the prednisone-MTX group, suggesting that the protective effect against MTX side-effects outweighs AEs caused by prednisone during the observation period. One other trial compared MTX monotherapy to treatment with MTX + prednisone (6.25 mg daily on average) in RA patients during 12 months and showed a similar trend in the occurrence of MTX side-effects leading to discontinuation (5.5% versus 9.1%, *p* = 0.29) but this was not statistically significant, and no correction for MTX dose or disease activity was made [8].

In CAMERA-II, 10 mg prednisone was used throughout the 2-year study period without any relevant prednisone related toxicity. This is in accordance with a meta-analysis assessing 6 other clinical trials comparing prednisone (5-10 mg) to placebo for at least 2 years in RA patients which also showed limited prednisone related toxicity [9]. In the long-term follow-up (median 6.7 years) of this trial (where many patients eventually tapered prednisone) no relevant prednisone-related toxicity was found [10]. However, it remains unclear if tapering prednisone after a period of use would eliminate the protective effect or whether MTX would remain well tolerated due to e.g. habituation. If concomitant low-moderate dose prednisone needs to be continued long-term to achieve alleviation of MTX side-effects, the protective effect of prednisone should of course be weighed against its possible long-term toxicity and comorbidity, which remains a topic of discussion and has been extensively reviewed by others [11].

The anti-emetic effect of glucocorticoids has already been established, specifically for chemotherapy induced nausea. Several mechanisms of action have been described, including the anti-inflammatory properties, influence on the hypothalamic–pituitary–adrenal axis, effect on serotonin-signalling and interaction with the adrenoreceptor [12]. The mechanism responsible for elevation of liver enzymes during low-moderate dose MTX therapy, may be via folate depletion, since folate supplementation reduces rates of hepatotoxicity [13]. However, even with folate supplementation, elevation of liver enzymes remains frequent as shown in this study. Interestingly, treatment with glucocorticoids has also been shown to reduce elevated liver enzymes in some cases of hepatitis B infection, presumably due to its anti-inflammatory effect [14]. It can be hypothesized that MTX induces a low grade inflammatory response in the liver, leading to elevation of liver enzymes which can be inhibited by prednisone, but not by tocilizumab. Our results show that the protective effect of prednisone is mainly present in women, which could partly be explained by a higher clearance and apparent volume of distribution of prednisone in men [15].

This analysis suggests that addition of low-moderate dose prednisone to MTX therapy in newly diagnosed rheumatoid arthritis patients may alleviate MTX side-effects. This might specifically be of interest in patients where the maximum MTX dose is limited by side-effects, given that prednisone is tolerated well, and other measures to reduce side-effects (such as subcutaneous administration or addition of folic acid) have insufficient effect.

## Supplementary Information

**Additional file 1. Supplementary Table S1.** Model for occurrence of individual MTX side-effects in CAMERA-II trial. **Supplementary Tables S2. A.** Model for occurrence of any adverse event in CAMERA-II trial. **B.** Model for occurrence of any adverse event, not including MTX side-effects in CAMERA-II trial. **Supplementary Table S3.** Occurrence of adverse events in CAMERA-II trial. **Supplementary Table S4.** Model for occurrence of MTX side-effects in U-ACT-EARLY trial.

## Data Availability

The datasets used and/or analysed during the current study are available from the UMC Utrecht on reasonable request.
