# Supplementary material for: Concomitant prednisone may alleviate methotrexate side-effects in rheumatoid arthritis patients
Source: BMC Rheumatol. 2023 May 17;7:8. doi: 10.1186/s41927-023-00331-0 (PMC10189989; doi:10.1186/s41927-023-00331-0)
Supplement: Supplementary file 1 — Additional file 1. Supplementary Table S1. Model for occurrence of individual MTX side-effects in CAMERA-II trial. Supplementary Tables S2. A. Model for occurrence of any adverse event in CAMERA-II trial. B. Model for occurrence of any adverse event, not including MTX side-effects in CAMERA-II trial. Supplementary Table S3. Occurrence of adverse events in CAMERA-II trial. Supplementary Table S4. Model for occurrence of MTX side-effects in U-ACT-EARLY trial. [file 41927_2023_331_MOESM1_ESM.docx]

**Additional File 1**

Supplementary Table S1. Model for occurrence of individual MTX side-effects in CAMERA-II trial.

1. Occurrence of nausea

|  | **Overall** | | | **Females^a^** | | |
| --- | --- | --- | --- | --- | --- | --- |
|  | **OR** | **95% CI** | **P-value** | **OR** | **95% CI** | **P-value** |
| **Use of prednisone** | **0.46** | **0.26-0.83** | **0.009** | **0.31** | **0.16-0.59** | **<0.001** |
| Male sex | 0.26 | 0.13-0.52 | <0.001 | - | - | - |
| MTX dose (mg)^b^ | 1.02 | 1.00-1.04 | 0.033 | 1.01 | 0.99-1.00 | 0.243 |
| DAS28^c^ | 1.15 | 1.04-1.27 | 0.009 | 1.12 | 1.01-1.25 | 0.034 |
| Intercept | 0.08 | 0.04-0.17 | <0.001 | 0.12 | 0.06-0.23 | <0.001 |

1. Occurrence of elevated ALT/AST

|  | **OR** | **95% CI** | **P-value** |
| --- | --- | --- | --- |
| **Use of prednisone** | **0.29** | **0.17-0.49** | **<0.001** |
| MTX dose (mg)^b^ | 1.00 | 0.98-1.02 | 0.907 |
| DAS28^c^ | 1.00 | 0.88-1.14 | 0.975 |
| ALT^d^ at baseline (U/L) | 1.02 | 1.01-1.03 | <0.001 |
| AST^e^ at baseline (U/L) | 1.03 | 1.00-1.06 | 0.037 |
| Intercept | 0.03 | 0.01-0.06 | <0.001 |

a. Model for male sex did not run due to too few occurrences of nausea in this group. b. Methotrexate c. Disease Activity Score assessing 28 joints d. Alanine transaminase e. Aspartate transaminase

Supplementary Tables S2

A. Model for occurrence of any adverse event in CAMERA-II trial.

|  | **OR** | **95% CI** | **P-value** |
| --- | --- | --- | --- |
| **Use of prednisone** | **0.89^a^** | **0.72-1.11** | **0.303** |
| Male sex | 0.56 | 0.46-0.68 | <0.001 |
| RF^b^ positivity | 0.86 | 0.92-1.48 | 0.199 |
| MTX^c^ dose (mg) | 1.02 | 1.01-1.03 | <0.001 |
| DAS28-ESR^d^ | 1.19 | 1.13-1.26 | <0.001 |
| Leucocytes at baseline^e^ (10^9^/L) | 0.95 | 0.92-0.99 | 0.010 |
| Number of visits | 0.98 | 0.96-1.00 | 0.024 |
| Intercept | 0.58 | 0.31-1.09 | 0.088 |

B. Model for occurrence of any adverse event, not including MTX side-effects in CAMERA-II trial.

|  | **OR** | **95% CI** | **P-value** |
| --- | --- | --- | --- |
| **Use of prednisone** | **1.05^f^** | **0.83-1.34** | **0.673** |
| Male sex | 0.54 | 0.44-0.68 | <0.001 |
| RF^b^ positivity | 0.79 | 0.61-1.03 | 0.084 |
| MTX^c^ dose (mg) | 1.20 | 1.14-1.27 | <0.001 |
| DAS28-ESR^d^ | 1.02 | 1.01-1.03 | <0.001 |
| Leucocytes at baseline^e^ (10^9^/L) | 0.98 | 0.96-1.01 | 0.145 |
| Number of visits | 0.96 | 0.92-1.00 | 0.050 |
| Intercept | 0.30 | 0.15-0.61 | 0.001 |

a. Corresponding relative risk: 0.92, CI: 0.78-1.08 b. Rheumatoid Factor c. Methotrexate d. Disease Activity Score assessing 28 joints e. In the analysis we explored correction for baseline hemoglobin, leukocytes, platelets, ALT/AST and creatinine, since values outside normal ranges were reported as adverse event. f, Correspongding relative risk: 1.04, CI: 0.86-1.25.

Supplementary Table S3. Occurrence of adverse events in CAMERA-II trial.

|  | **MTX^a^ + prednisone** | | **MTX^a^ Mono** | |
| --- | --- | --- | --- | --- |
|  | **(N=117; 2187 visits)** | | **(N=119; 2421 visits)** | |
| **Serious adverse event** |  |  |  |  |
| Death | 1 | (0%) | 0 | (0%) |
| Hospitalization | 1 | (0%) | 5 | (0.2%) |
| **Infection** |  |  |  |  |
| Antibiotics needed | 42 | (1.9%) | 62 | (2.6%) |
| **Gastro-intestinal (GI)** |  |  |  |  |
| Nausea | 50 | (2.3%) | 152 | (6.3%) |
| Vomiting | 7 | (0.3%) | 8 | (0.3%) |
| Abdominal pain | 13 | (0.6%) | 17 | (0.7%) |
| Decreased appetite | 1 | (0%) | 4 | (0.2%) |
| Anorexia | 0 | (0%) | 2 | (0.1%) |
| Diarrhea | 16 | (0.7%) | 16 | (0.7%) |
| Subjective GI complaints | 72 | (3.3%) | 51 | (2.1%) |
| Other | 12 | (0.5%) | 2 | (0.1%) |
| **Mucocutaneous** |  |  |  |  |
| Stomatitis | 3 | (0.1%) | 2 | (0.1%) |
| Oral ulcers | 15 | (0.7%) | 35 | (1.4%) |
| Itch | 18 | (0.8%) | 24 | (1%) |
| Hair loss | 40 | (1.8%) | 60 | (2.5%) |
| Other | 49 | (2.2%) | 52 | (2.1%) |
| **Nervous system** |  |  |  |  |
| Headache | 58 | (2.7%) | 66 | (2.7%) |
| Dizziness | 51 | (2.3%) | 45 | (1.9%) |
| Mood changes | 11 | (0.5%) | 23 | (1%) |
| Blurry vision | 34 | (1.6%) | 30 | (1.2%) |
| Dry eyes | 24 | (1.1%) | 13 | (0.5%) |
| Decreased hearing | 7 | (0.3%) | 4 | (0.2%) |
| Other | 62 | (2.8%) | 97 | (4%) |
| **Metabolic** |  |  |  |  |
| *Creatinine level increase (> ULN^b^)* | *2* |  | *1* |  |
| *Hypertension (> 140 or > 90 mmHg)* | *11* |  | *18* |  |
| *Diabetes (>11 mM non-fasting glucose)* | *1* |  | *1* |  |
| **Liver** |  |  |  |  |
| Elevated ALT and/or AST^c^ (>2x ULN^b^) | 48 | (2.2%) | 84 | (3.5%) |
| Other | 3 | (0.1%) | 8 | (0.3%) |
| **Hematologic** |  |  |  |  |
| Anemia | 21 | (1%) | 43 | (1.8%) |
| Leukopenia | 1 | (0%) | 18 | (0.7%) |
| Thrombocytopenia | 2 | (0.1%) | 0 | (0%) |
| **Pulmonary** |  |  |  |  |
| Coughing | 33 | (1.5%) | 21 | (0.9%) |
| Dyspnea | 9 | (0.4%) | 2 | (0.1%) |
| *Pneumonitis* | 1 | (0%) | 0 | (0%) |
| Subjective pulmonary complaints | 4 | (0.2%) | 5 | (0.2%) |
| **Bone** |  |  |  |  |
| *Fracture* | 1 | (0%) | 0 | (0%) |
| *Osteoporosis* | 0 | (0%) | 0 | (0%) |

Table represents number of visits at which adverse events (AEs) were reported. Persistent or recurrent AEs could be reported multiple times by one patient, except for conditions in italics (reported only once per patient). a. Methotrexate. b. Upper limit of normal c. Alanine transaminase / aspartate transaminase

Supplementary Table S4. Model for occurrence of MTX side-effects in U-ACT-EARLY trial.

|  | **OR** | **95% CI** | **P-value** |
| --- | --- | --- | --- |
| **Use of tocilizumab** | **1.05^a^** | **0.61-1.80** | **0.872** |
| Age (years) | 0.98 | 0.96-0.99 | 0.005 |
| MTX dose (mg)^b^ | 1.02 | 0.99-1.05 | 0.128 |
| DAS28-ESR^c^ | 1.12 | 0.97-1.29 | 0.120 |
| Intercept | 0.13 | 0.04-0.35 | <0.001 |

a. Corresponding relative risk: 1.02, CI: 0.79-1.23. b. Methotrexate c. Disease Activity Score assessing 28 joints
